# Underwater Inherent Optical Properties Estimation Using a Depth Aided Deep Neural Network

**DOI:** 10.1155/2017/8351232

**Published:** 2017-11-15

**Authors:** Zhibin Yu, Yubo Wang, Bing Zheng, Haiyong Zheng, Nan Wang, Zhaorui Gu

**Affiliations:** ^1^Department of Electronic Engineering, College of Information Science and Engineering, Ocean University of China, Qingdao, China; ^2^School of Life Science and Technology, Xidian University, Xi'an, China

## Abstract

Underwater inherent optical properties (IOPs) are the fundamental clues to many research fields such as marine optics, marine biology, and underwater vision. Currently, beam transmissometers and optical sensors are considered as the ideal IOPs measuring methods. But these methods are inflexible and expensive to be deployed. To overcome this problem, we aim to develop a novel measuring method using only a single underwater image with the help of deep artificial neural network. The power of artificial neural network has been proved in image processing and computer vision fields with deep learning technology. However, image-based IOPs estimation is a quite different and challenging task. Unlike the traditional applications such as image classification or localization, IOP estimation looks at the transparency of the water between the camera and the target objects to estimate multiple optical properties simultaneously. In this paper, we propose a novel Depth Aided (DA) deep neural network structure for IOPs estimation based on a single RGB image that is even noisy. The imaging depth information is considered as an aided input to help our model make better decision.

## 1. Introduction

Light always plays an important role in physics, chemistry, and biology of oceans research. The process of the light transmission in the seawater is the foundation of ocean optical research. And the optical properties are the key to describe the light transmission process. The wavelength of the visible light is widely spread from 400 nm to 700 nm. The optical properties of the medium are crucial for the further research of underwater vision, marine organism, pollution detection, and other ocean research areas. The optical properties of ocean can be roughly classified as inherent optical properties (IOPs) and apparent optical properties (AOPs). Important IOPs contain spectral absorption coefficient, *a*(*λ*), scattering coefficients, *b*(*λ*), and attenuation coefficient, *c*(*λ*). For a certain wavelength *λ*, these three properties can be simply described as

(1) $$\begin{array}{c} c\left(\;\lambda \;\right)=a\left(\;\lambda \;\right)+b\left(\;\lambda \;\right). \end{array}$$

IOPs only correlate with medium itself and are irrelevant to the ambient light field or its geometric distribution. Measuring these coefficients is fundamental and important to ocean optical research. Beam transmissometers, such as ac-spectra (AC-S) produced by Wetlabs, are the most commonly used devices for IOPs measurement [1–3]. However, its inconvenient to place such an equipment because of the high price and the limited volume of underwater IOPs measured by the device. Moreover, most of the researchers consider the water as homogeneous medium when measuring IOPs [4–7]. Actually, a slight turbulence caused by robots, marine pollution, or organisms may lead to an inhomogeneous medium. But the beam transmissometers can only detect the IOPs in the surrounding area. In such a case, an underwater camera with a real-time system will be more flexible to capture efficient information. Besides, there are some other ways to deduct IOPs based on AOPs [8, 9].

AOPs are those properties that depend both on the medium (the IOPs) and on the geometric structure of the radiation distribution. AOPs can be measured by remote sensing. And many researchers prefer to measure IOPs with transmissometers as the ground truth to verify their AOPs based deduction. But such deduction is not accurate enough. On one hand, data based on remote sensing is obtained from satellites or airplane. The detailed information may be omitted. On the other hand, remote sensing cannot investigate the IOPs of undersea. AOPs based on remote sensing can only tell us the IOPs distribution of the surface water. Research also shows that the depth of water may bring difficulty when we want to calculate IOPs [10].

Underwater images are always with scattering and absorption. Because of these reasons, underwater images are always blurred because of different light field and IOPs, which may bring difficulty for us to build an accurate physical model [11]. However, there are many researchers showing that they can recover an underwater image with IOPs [12–14] and physical models [15]. That means an underwater image contains plenty of IOPs information. That is why we can restore images with correct IOPs and suitable physical models. If we can estimate IOPs with a single image, it would be much convenient to measure IOPs undersea. Unlike the most of underwater image restoration tasks which aim to reduce the noise caused by scattering and absorption effect [16], the image noisy information will help us to deduct IOPs.

Since Hinton and his colleges proposed the deep learning concept from 2006 [17], deep neural network becomes more and more popular these years. Neural network models are proven not only on computer vision, but also on symmetric recognition, image quality assessment, image restoration, and even optical flow processing [18–23]. Thus, we consider if it is possible to use deep neural network for analyzing underwater images to estimate IOPs in this study. Besides, if we want to build an end-to-end system with an image as inputs and IOPs as outputs, deep neural network is a suitable candidate to connect them together. In this paper, we used AC-S to provide 156 IOPs (78 attenuation coefficients and 78 absorption coefficients) as the ground truth for the neural network training.

## 2. Depth Aided Deep Neural Networks for IOPs Estimation

**Figure 1:**
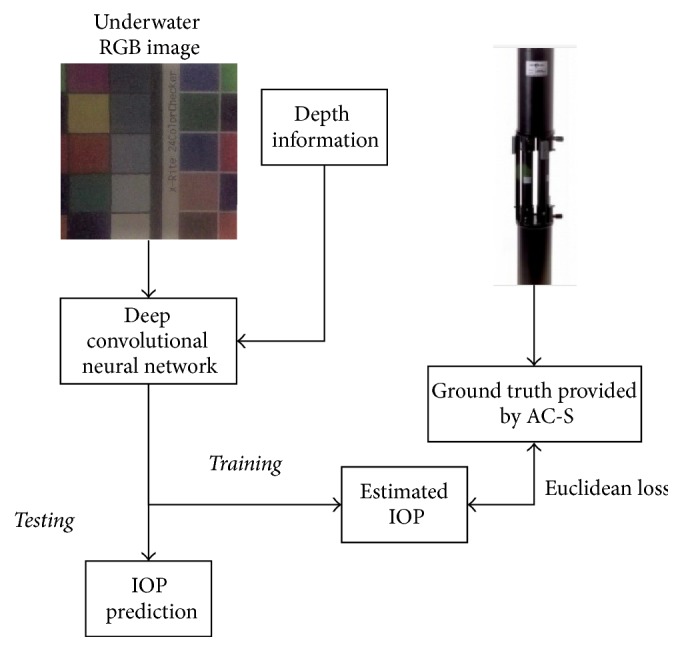
The framework of our experiment.

The framework of our system can be found in Figure 1. We used a color calibration board as the target underwater object and captured its RGB images with a video camera. We also measured the distance between the board and the camera as depth information. The depth information is used as an aided input to the deep convolutional neural network. Convolutional neural network (CNN), which is developed by Lecun and Bengio [24] in 1995, is a powerful model especially in computer vision research. CNN is improved by Krizhevsky and his colleges in 2012 as AlexNet [25]. We follow their idea to build our model for underwater image analysis. However, our inputs and outputs are different from their work.

Deep neural network can not only recognize what kind of the target object is, but also understand how much blur an image is. But the water quality is not the only factor which causes the image blur. In physical point of view, distance between the camera and the target is also an important factor. The underwater images would be blur if the water quality is low, and the images would also be unclear if the target object is far away from the camera. That is the reason why we considered depth information of the target object as another useful feature.

Unlike some common neural network application tasks such as image classification, IOPs including multiple coefficients are not binary values. Thus, softmax activation function which is usually used for image classification tasks [26–28] in the last layer cannot be used for our goal. Instead, we applied min–max normalization and Euclidean loss function for IOPs regression:

(2) E=12N∑i=1Nyi−yi∗2,

(3) yi∗=yi,original∗−ymin⁡ymax⁡−ymin⁡,

where *y*_*i*,original_^*∗*^ is the *i*_th_ desired IOP coefficient measured by AC-S, *y*_*i*_^*∗*^ is the IOP coefficient after min–max normalization, *y*_*i*_ is the *i*_th_ estimated IOPs by deep neural network, and *N* is the number of IOPs. The AC-S employed can provide 78 absorption and 78 attenuation coefficients. Hence, we have *N* = 78 × 2 = 156. Equation (3) shows the detail of min–max normalization which is used for depth normalization. We also use this method to normalize the depth information.

**Figure 2:**
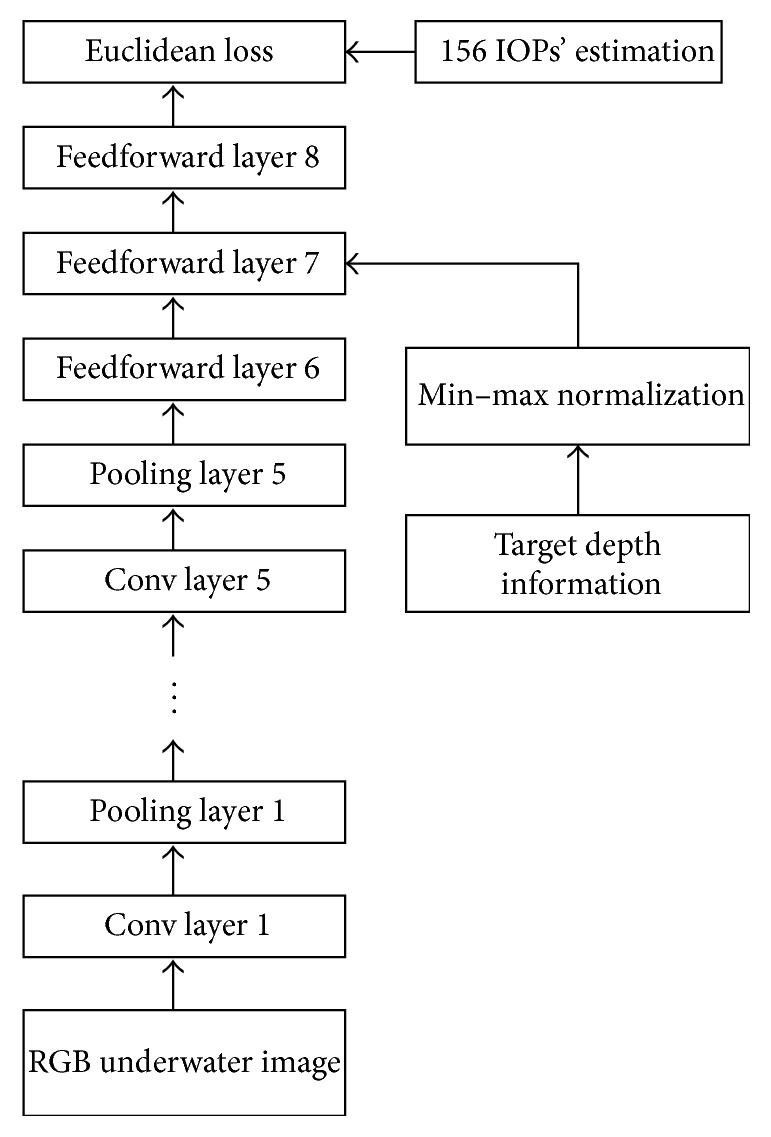
The Depth Aided (DA) deep neural network structure.

We design the Depth Aided (DA) neural network model for IOPs estimation as shown in Figure 2. The AC-S can provide 78 attenuation coefficients and 78 absorption coefficients from 400 nm to 730 nm as network output labels when we capture RGB images. These IOPs are used as the targets of the AlexNet. Because our target object is a flat board, depth information is considered as a single number. Thus there is no need to put the depth information into convolution layer. So we set the depth information as an aided input in the feedforward layer 7. The weights between depth information and feedforward layer 7 are fully connected. Error backpropagation algorithm, stochastic gradient descendent (SGD), and dropout algorithms [29] are used for evolving this network. The feedforward connections of each neurons in feedforward layer 7 can be described as

(4) $$\begin{array}{c} y_{i}=f\left(\;u_{j}\;\right)=f\left(\;w_{f}x_{f}+w_{d}x_{d}+b\;\right), \end{array}$$

where *y*_*j*_ and *u*_*j*_ are the *j*_th_ neuron postsynaptic and presynaptic value of the feedforward layer 7, respectively; *x*_*f*_ is the neural inputs from the feedforward layer 6; *w*_*f*_ is the weights between layer 6 and layer 7, *x*_*d*_ is the depth information; *w*_*d*_ is the weight in depth information; *b* is the bias; and *f*() is the activation function. The network weights updating can follow the error backpropagation rule as

(5) ∂E∂wij=∂E∂ujyi,Δwt+1=μΔwt−η∂E∂wt+1−ξwt,

where *w*_*ij*_ represents the weight from the *i*_th_ neuron to the *j*_th_ neuron, *E* is the error function defined in (2), Δ*w*(*t* + 1) is the weight updating value in the (*t* + 1)_th_ iteration, *μ* is the momentum, *η* is the learning rate, and *ξ* is the weight decay rate which can prevent overfitting.

**Figure 3:**
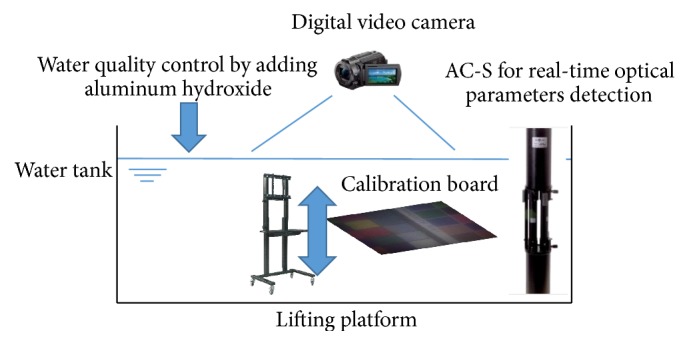
The framework of our experiment.

**Figure 4:**
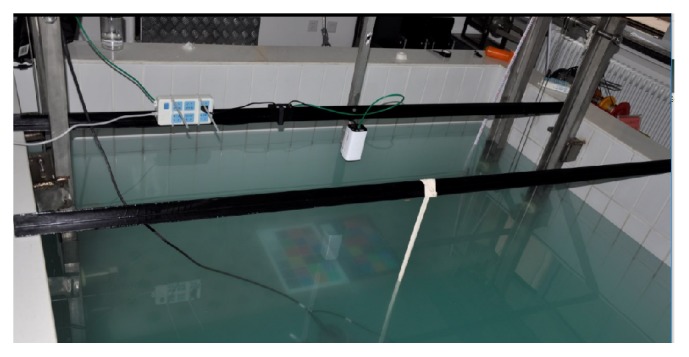
Experiment environment.

Our datasets are collected in a large water tank as shown in Figures 3 and 4. We put a lifting platform inside the tank to hold the color calibration board. The digital camera is just above the water. The lifting platform can guarantee the board to always be inside the water and vertical to the camera. And it can also change the distance between the color board and the camera accurately. After we get enough data with different distances, we added the aluminium hydroxide into the water to change the water qualities and collect the data again. Meanwhile, we also use AC-S in the water to measure the real-time IOPs as ground truth. We did not use any additional light field in this experiment except indoor diffuse refection.

The data we collected are listed in Tables 1 and 2. Due to the size of the employed water tank (3.6 m (length) × 2.0 m (width) × 1.2 m (depth)), the precise concentration of aluminium hydroxide cannot be directly measured. However, we could estimate the concentration of aluminium hydroxide by using the volume of water filled in the tank and the weight of aluminium hydroxide added for each image collection. The results were given in Table 2. To ensure uniformity of aluminium hydroxide distribution, we used a circulating pump to stir the water before image collection started. Yet, it can be noticed that the average attenuation and absorption of image pack (4) look lower than the value obtained for image pack (3). The reason for that is the first 3 image packs and the remaining 6 image packs were taken on two consecutive days; some aluminium hydroxide settled at the bottom still standing after 10 hours.

We collected 3 groups of datasets. Please note that we do not add any aluminium hydroxide into the water when we take photos in image pack (1). Datasets A and B were taken under similar environments but at different time periods. Dataset C was taken with different distances and different IOPs. Each image pack was captured with a digital video camera during a short period. Lots of researchers used IOPs under 520 nm wavelength as reference properties [30, 31]. The average value of attenuation and absorption coefficients at 520 nm wavelength in each image pack can be found in Table 1. The frame rate of this camera is 25 frames per second. After we take enough images under a certain depth we can modify the distance between the camera and the board by adjusting the lifting platform. When we got enough photos in one pack, we modified the water IOPs by adding aluminium hydroxide and then started capturing the next image pack. The IOPs in one image pack are similar. But we still use the real-time results provided by AC-S as the training label of deep convolutional neural networks. Our camera type is Hikvision 2ZCN3007. It used a 1/2.8′′ Progressive Scan CMOS sensor. The camera can provide videos with resolution of 1080 p. We used the raw RGB camera pictures (1920*∗*1080 pixels) and chose the center part as the region of interest (800*∗*800 pixels) as shown in Figure 6. And then we resized them into 200*∗*200 for network training. The input of the neural network used 3 channels for RGB format. We collected images in the daytime. The camera used an automatic exposure system to record images. There is no other additional light source during our experiments except the diffuse reflection. No additional image preprocessing methods are used.

**Figure 5:**
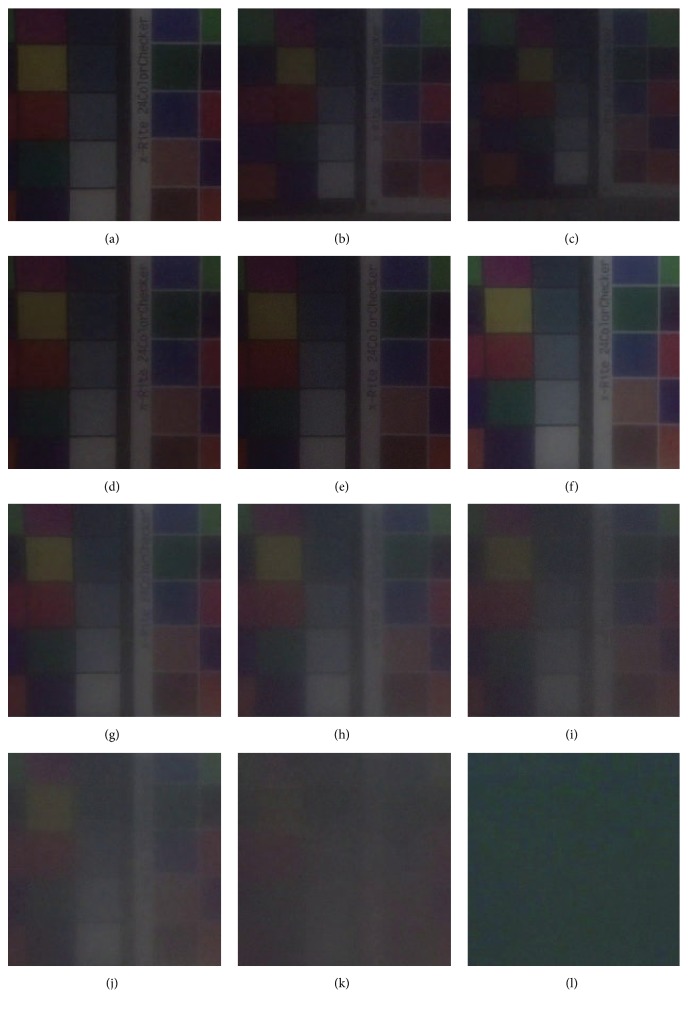
Images captured under different situations. (a)–(c) are captured without any aluminium hydroxide (image pack (1)) under 460 mm, 560 mm, and 660 mm. (d)–(l) are captured under 460 mm corresponding to image packs (2)–(10).

**Figure 6:**
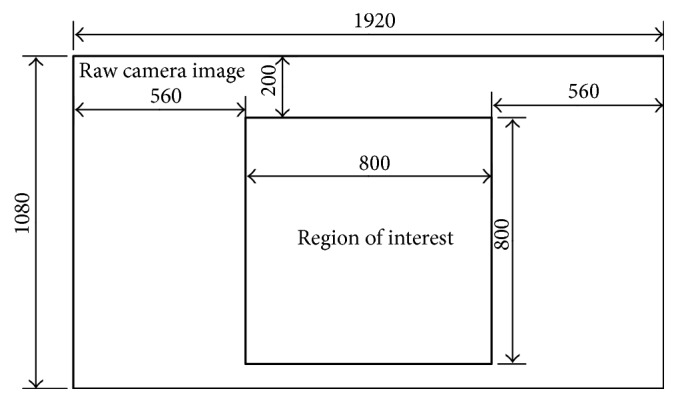
The ROI position.

The sample images of 10 different image packs are displayed in Figure 5. Figures 5(a)–5(c) are captured in pack (1) under 460 mm, 560 mm, and 660 mm, respectively, and Figures 5(d)–5(l) are captured from pack (2) to pack (10) under the same distance (460 mm). The overview of IOPs estimation results can be found in Table 3. We use a single GTX1070 graphic card and Intel i7-6700 to train these networks. We set the learning rate as 0.0001. We use 3 kinds of deep neural network for IOPs estimation evaluation. We waited enough epochs until these networks converged. Cifar-Net, which is improved based on LeNet-5 [22], is used as benchmark for this experiment. Although we waited 100,000 epochs (about 1 hour), the results based on Cifar-Net are still poor even in training set. AlexNet, which costs us about 3 hours on training until we reach 30,000 epochs, performs better. And we get the minimum Euclidean loss if we consider the depth information. Lower loss means estimated IOPs are closer to the ground truth. That means depth information is helpful especially in clean medium. The DA Net costs about 3 and a half hours for 30,000 epochs. The training speed of our model is a little slower than AlexNet, but with better performance.

**Figure 7:**
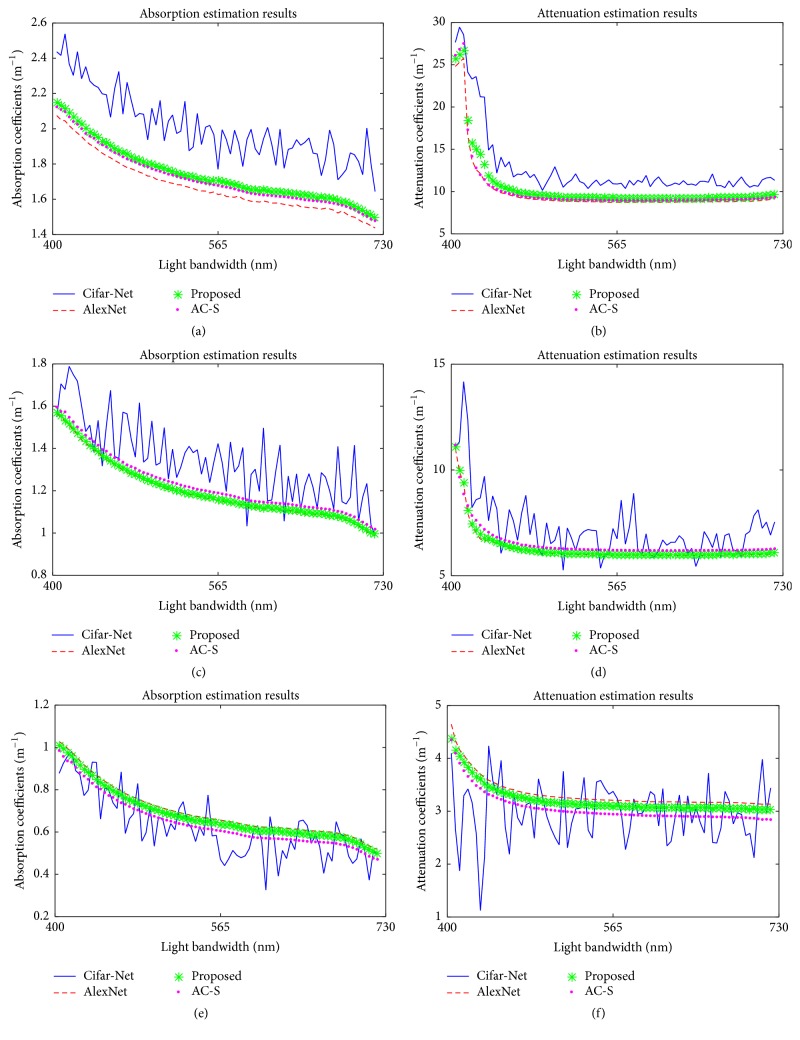
IOPs estimation results. (a)–(f) show the IOPs estimation results based on 3 typical images corresponding to high, medium, and low turbidity. (a) and (b) show the attenuation and absorption coefficients regression results based on Figure 5(i). (c) and (d) show the coefficients regression results based on Figure 5(f). (e) and (f) show the coefficients regression results based on Figure 5(d).

The detail of IOPs estimation results can be found in Figure 7. We choose 3 typical RGB images representing images captured in high, medium, and low turbidity, respectively. The performance of Cifar-Net is shown using blue lines; the regression curve of AlexNet is displayed with red dashed lines; the DA Net is shown with green asterisks and the ground truth provided by AC-S is represented using purple dot lines. In high turbidity case, both AlexNet and our method perform well on attenuation regression. A small amount error existed in blue-purple band (400–450 nm). Our method performs better than AlexNet in both absorption and attenuation coefficients regression task. In medium and low turbidity case, the curves of the DA Net get closer to the ground truth comparing with AlexNet. Although Cifar-Nets show a generally right regression result on three cases, its performance is much lower than the other two methods.

## 3. Discussion and Conclusion

In summary, we propose a DA deep neural network for IOPs estimation method based on a single RGBD image with a DA deep neural network. We argue that an underwater image contains enough IOPs information that is even noisy. So we are able to deduct IOPs on a single RGBD image with a suitable system. Comparing with traditional methods based on transmissometers, our method can archive enough accuracy but cost-effectively and more flexibly than traditional devices. Our method is able to predict both attenuation and absorption coefficients of the medium simultaneously. The experimental results in Table 3 show that even a single RGB image seems enough for IOPs estimation with deep learning technologies. We can get better estimation results if we consider depth information as an aided input.

In our experiment, we did not consider any complex light field conditions and target objects with complex shape case. These factors may bring difficulty to measure IOPs when we want to put this system in an opening environment. Fortunately, research on deep neural network shows that it is possible to estimate a depth map on a complex target object and even under different light fields with a single RGB image [32, 33]. That may be a possible solution for us to improve our model. On the other hand, back-scattering coefficients, which cannot be measured by AC-S, are very important to build an underwater image recovering model. How to estimate back-scattering coefficients is another challenge. We wish to leave these two parts in our future work.

## Figures and Tables

**Table 1:** Datasets description.

Datasets	Distance (mm)	Images	Image pack
Dataset A	500,600,700	2100	4–10
Dataset B	500,600,700	2100	4–10
Dataset C	460,560,660,760	1200	1–3

**Table 2:** IOPs description.

Image pack	Image number	Avg. attenuation (m^−1^)	Avg. absorption (m^−1^)	Aluminium hydroxide (g/m^3^)
(1)	400	2.9380	0.6392	0
(2)	400	3.2664	0.6596	4.24
(3)	400	3.2725	0.6905	6.37
(4)	600	3.0048	0.6505	6.37
(5)	600	4.1257	0.8627	8.53
(6)	600	5.0092	1.0213	10.76
(7)	600	5.8371	1.1759	12.58
(8)	600	6.2485	1.2357	17.24
(9)	600	9.0333	1.7242	25.61
(10)	600	11.7797	2.0721	34.89

**Table 3:** IOPs estimation results.

Training set	Test set	Euclidean loss	Network
A	A	1.23	Cifar-Net
A	A	2.67	Cifar-Net
A	A	2.70	Cifar-Net
A	B	0.047	AlexNet
A	B	0.232	AlexNet
A	B	0.1532	AlexNet
A	C	0.032	DA Net
A	C	0.1996	DA Net
A	C	0.056	DA Net
